# Effects of garlic, onion, and apple cider vinegar as a herbal mixture on performance and blood traits of broilers inoculated with chicken infectious anemia virus

**DOI:** 10.1016/j.heliyon.2023.e17768

**Published:** 2023-06-28

**Authors:** Zana Azeez Abdulkareem, Nihayat Ibrahim Mohammed, Asrin Abdollahi, Omer Rasool Ahmed, Osama Rahman Ghaffar, Hawkar Azad Khdir, Dashty Akram Salam, Sarhang Ahmad Aziz, Mustafa Mama Mustafa, Warzer Mohammed Mustafa, Zaniar Ali Abas, Omed Idrees Abid

**Affiliations:** a Department of Animal Resources, Collage of Agricultural Engineering Sciences, University of Raparin, Ranya, Sulaymaniyah, 46012, Iraq; b Department of Animal Science, University of Kurdistan, Sanandaj, 66177-15175, Iran; c Central Veterinary Laboratory, Sulaymaniyah, 46001, Iraq

**Keywords:** Anemia, Apple cider vinegar, Garlic, Mortality, Onion

## Abstract

This study assessed the effects of a herbal mixture (HM) to protect poultry against chicken infectious anemia (CIA) and to modulate the adverse effects of this virus on performance, mortality, blood profile, white blood cells (WBCs) count, liver enzymes, liver histopathology, and intestinal morphology. Therefore, 240 one-day-old male broiler chicks (Ross 308) were divided into four experimental groups, with six replicates and ten chicks per group. The experimental groups consisted of a control group and groups with 2.5%, 5%, and 7.5% HM, all based on corn-soybean meal. All chicks were inoculated with the CIA virus (CIAV) on day 7. The results showed that supplementation of 2.5% of HM to broiler diet increased feed intake (FI) (P < 0.05) and also increased body weight (BW) and weight gain (WG) slightly (P > 0.05). Adding 7.5% HM caused a reversible decrease in FI, BW, and WG and increased FCR. Compared with the control group, mortality rates declined with an additional dose of HM in CIAV-infected chickens. HM supplementation in the diet of CIAV-infected chickens increased hematocrit (HCT), hemoglobin (Hb), and mean corpuscular volume (MCV) and decreased mean corpuscular hemoglobin concentration (MCHC) compared to the control (P < 0.05). Lymphocyte percentage and lymphocyte/heterophile ratio increased in HM-supplemented groups, especially at 2.5% (P < 0.05), and heterophile and granulocyte percentages were reduced (P < 0.05). Liver enzyme alkaline phosphatase (ALP) and liver steatosis declined in the 2.5% HM-treated group compared to the control (P < 0.05). It was concluded that adding 2.5% of the HM to the CIAV-infected broiler’s diet did not negatively affect chicken performance. In addition to its hypolipidemic effects, it could prevent HCT and Hb from decreasing in chicks infected with CIAV and positively affect leukocyte types and liver enzymes. Interestingly, an additional dose of HM in the diet of the CIAV-infected broilers reduced mortality. Therefore, adding 2.5% of HM could prevent the adverse effects of CIA on hematological traits in broiler chicken flocks without adverse effects on performance.

## 1 Introduction

Chicken infectious anemia is a vital poultry disease caused by the chicken infectious anemia virus (CIAV), which belongs to the genus Gyrovirus in the Circoviridae family. It has spread rapidly worldwide since CIAV's initial isolation in Japan [1]. CIAV can spread vertically or horizontally, with the most crucial vertical transmission occurring from parents to young chicks through hatching eggs [2]. Young chicks suffer from infectious anemia caused by CIAV, a potent immunosuppressive agent. The virus predominantly affects lymphoid and hematopoietic tissues, making them more vulnerable to secondary infections and suppressing the immune system's ability to fight diseases and perform well under environmental circumstances [3,4]. Because CIAV spreads continuously among flocks of chickens, its eradication is challenging [5]. Immunomodulatory techniques using haematinics and immunostimulants have been proposed to control clinical pathologies [3,6,7].

Herbal immunomodulation is a traditional treatment known worldwide for its preventative and therapeutic effects against various diseases, including viral infections [8]. Various medicinal plants play an important role in poultry nutrition due to their beneficial effects [9]. The active substances in medicinal plants play an essential role as alternatives to antibiotics and vaccinations, boosting immunity, decreasing mortality, affecting gastrointestinal enzymatic activity, and enhancing nutrient digestion [10]. The combined use of medicinal plants as a dietary additive works better in modulating the adverse effects of CIAV.

Onion (*Allium cepa* L.) belongs to the *Liliaceae* family and contains organic sulfides, including s-propyl cysteine sulfoxides, s-methyl-cysteine sulfoxides, *trans*-*s*-(1-propenyl) cysteine sulfoxides, and cyclo allicin. It also contains flavonoids, phenolic acids, and sterols, including cholesterol, b-sitosterol, saponins, and stigma sterol [11]. Onions have numerous health benefits including antibacterial, antiviral, antiparasitic, and antifungal properties [12]. Dietary intake of dried onions significantly reduced serum cholesterol [13]. Goodarzi et al. [14] reported that onion extracts positively affect the growth performance of broiler chickens. Garlic (*Allium sativum*) is a well-known spice and herbal medicine used to prevent and treat various diseases [15]. The sulfur-containing chemicals allin, diallyl sulfides, and allicin, which are the bioactive components of garlic, have antibacterial, antifungal, antiparasitic, antiviral, antioxidant, antithrombotic, anticancer, and vasodilator properties. Garlic has been shown to decrease serum and liver cholesterol [16]. Broiler chicken performance was enhanced by dietary garlic supplementation. For approximately 50 years, garlic has been used to increase poultry growth and as a growth-promoting antibiotic [17]. Apple cider vinegar is produced naturally during apple fermentation. It contains polyphenols with antioxidant properties, organic acids such as acetic acid (3–9%), vitamins, and minerals [18]. Supplementation with apple cider vinegar strengthens the body's defense against infections, improves immunity, and maintains the acid-base balance [19].

In addition to the above-mentioned medicinal properties, onion and garlic flavonoids have a significant inhibitory effect on viral replication, and the phytochemicals in these plants have been found to prevent viruses from forming proteins and genetic material [[20], [21], [22]]. Organosulfur compounds of *Allium* spp. are potent antiviral agents which reduce viral activity by blocking attachment, fusion, and entry of viruses into the host cell [[23], [24], [25], [26]]. Garlic also increased erythropoiesis in an erythropoietin-independent manner in the spleen [27]. Ujike et al. [28] and Takatera et al. [29] have reported that apple cider vinegar and citric acid, the primary acids in vinegar, increase iron absorption in the intestine. Ousaaid et al. [30] reported that apple cider vinegar reduces the hemolytic effect of phenylhydrazine in rats with anemia. In addition to these medicinal properties, accessibility to most areas leads to onion, garlic, and apple cider vinegar being candidates for herbal immuno-modulation. To take advantage of these three components, we used them as a herbal mixture to evaluate their effects against CIAV in broiler chickens.

Polyherbal medications may be safer and more economical for the prevention and treatment of immunosuppressive viruses and other infections. This study aimed to evaluate the effects of a herbal mixture (HM) on CIA in broiler chickens. Therefore, the effects of HM (garlic, onion, and apple cider vinegar) supplementation on the performance, carcass composition, blood profile, serum biochemical traits, liver enzymes, and intestinal morphology were evaluated.

## 2 Materials and methods

### 2.1 Herbal mixture (HM) preparation and analysis

Garlic tubers and onions used for herbal mixture preparation were purchased from a local farmer, dried, and powdered. Garlic, onion, and apple cider vinegar powders were mixed at a ratio of 1:1:0.2 (w:w:w). The HM chemical compositions, such as crude protein, ether extract, crude fiber, and ash content, were determined according to AOAC-1995 [31].

### 2.2 Ethanolic extraction of herbal mixture

Air-dried HM powder (20 g) was extracted (using a maceration method) by adding 200 mL of ethanol (99.8%) and shaking at 85 rpm and 25 °C for 6 h. The extract was filtered through Whatman No. 1 filter paper using a Buchner funnel. The filtrate was evaporated to dryness under reduced pressure in a rotatory vacuum evaporator (Heidolph, North America, Wood Dale, IL, USA) using a method described by Nassiri‐Asl and Hosseinzadeh [32]. The dried extracts were kept in the dark at 4 °C until GC-MS analysis.

GC-MS analysis was carried out using an Agilent 7890A Gas Chromatograph equipped with an electron impact quadrupole MD 800 mass spectrometer detector and a 19,095–400 fused silica column (30 m length and 0.25 mm inner diameter). The stationary phase has a thickness of 0.25 μm. The temperature of the initial injection column was 35 °C for 2.50 min, and temperature gradually increased to 280 °C for 20 min in 7 °C increments. The identities of the volatile oil components were established from their GC Kovats retention indices and mass spectra using computer matching with a mass spectra library (Adams, NIST, and Wiley).

### 2.3 Birds, housing, and experimental diets

All experiments were conducted at the Department of Animal Resources, College of Agricultural Engineering Sciences, University of Raparin, Ranya, Sulaymaniyah, Iraq. The University of Raparin Animal Care and Ethical Committee and complied with international guidelines [33], evaluated and approved all animal care and use procedures. In total, 240 one-day-old Ross 308 male broilers were randomly assigned to four treatments. Each treatment consisted of six replicates with ten birds per replicate, reared in 24 pens in a completely randomized design.

The experimental period was divided into three feeding phases: starter (1–10 days), grower (11–25 days), and finisher (26–35 days). The Ross 308 strain recommendations were used to formulate the diets. The experimental groups consisted of a control group and groups with 2.5%, 5%, and 7.5% HM, all based on corn-soybean meal. The composition and nutrient contents of the experimental diets are presented in Table 1, Table 2, Table 3. The birds were raised on wood shavings for bedding, had ad libitum access to water, and were provided pellets as food. A complete lighting program was used for the first 48 h, followed by 23 h of light and an hour of darkness for the remainder of the experiment.

**Table 1:** Ingredients and nutrients composition of experimental diets (1–10 days).

Ingredient (%)	Control	Herbal Mixture (%)		
		2.5	5	7.5
Corn grain	49.67	47.80	45.93	44.05
Soy bean meal (41% CP)	45.36	44.74	44.12	43.50
Soybean oil	0.00	0.00	0.00	0.00
Dicalcium phosphate	0.91	0.90	0.90	0.89
CaCO_3_	1.00	1.00	1.00	1.00
Common Salt	0.28	0.28	0.27	0.27
Premix concentrate^a^	2.50	2.50	2.50	2.50
DL-Methionine	0.27	0.27	0.28	0.28
Herbal mixture	0.00	2.50	5.00	7.50
**Calculated analysis**				
Crude protein, %	23	23	23	23
ME, kcal/kg	3000	3000	3000	3000
Calcium, %	0.96	0.96	0.96	0.96
Na, %	0.18	0.18	0.18	0.18
K, %	1.06	1.07	1.08	1.09
Cl, %	0.26	0.26	0.26	0.26
Total Phos, %	0.78	0.79	0.81	0.83
Avail Phos, %	0.51	0.52	0.52	0.53
Cysteine, %	0.37	0.37	0.37	0.37
Lysine, %	1.49	1.48	1.47	1.47
Methionine, %	0.67	0.67	0.67	0.68
TSAA, %	1.00	1.00	1.00	1.00
Threonine, %	0.98	0.97	0.97	0.96
Crude Fiber, %	1.69	1.73	1.78	1.83
Linoleic acid,%	1.27	1.23	1.19	1.14
**Analyzed compositions**				
Dry matter, %	92.99	92.83	93.02	93.98
Crude protein, %	23.21	23.01	23.19	23.12
Crude Fiber, %	1.41	1.43	1.63	2.21
Ash,%	7.21	6.97	7.15	7.04

Abbreviation: CP, crude protein; ME, Metabolisable energy; Phos, Phosapate; TSAA, total sulfur amino acid.

a Provided per kg of diet: Protein, 18%; CaCO_3_, 19%; Monocalcium phosphate, 7%; NaCl, 4.5%; Methionine, 2%; Lysine, 4%; Threonine, 1%; Choline chloride, 1%; Iron (sulphate), 2800 mg; Zinc (oxide), 2480 mg; Manganese (oxide), 3600 mg; Copper (sulphate), 240 mg; Iodine (IK), 60 mg; Selenium (SeNa), 14 mg; Cobalt (sulphate), 14 mg; Magnesium (oxide), 800 mg; Antioxidant (BHT), 32 mg; Vitamin A, 420,000 IU; Vitamin D_3_, 80,000 IU; Vitamin E, 800 mg; Vitamin C, 40 mg; Vitamin K_3_, 80 mg; Vitamin B_1_, 80 mg; Vitamin B_2_, 200 mg; Vitamin B_6_, 80 mg; Vitamin B_12_, 0.6 mcg; Biotine, 10.4 mg; Folic acid, 20 mg; Nicotinic acid, 400 mg; Pantothenic acid, 160 mg.

**Table 2:** Ingredients and nutrients composition of experimental diets (11–25 days).

Ingredient (%)	Control	Herbal Mixture (%)		
		2.5	5	7.5
Corn grain	54.67	52.80	50.92	49.05
Soy bean meal (41% CP)	40.80	40.18	39.55	38.93
Soybean oil	0.00	0.00	0.00	0.00
Dicalcium phosphate	0.55	0.55	0.55	0.54
CaCO_3_	1.00	1.00	1.00	1.00
Common Salt	0.27	0.27	0.27	0.27
Premix concentrate^a^	2.50	2.50	2.50	2.50
DL-Methionine	0.19	0.19	0.19	0.20
Herbal mixture	0.00	2.50	5.00	7.50
**Calculated analysis**				
Crude protein, %	21.5	21.5	21.5	21.5
ME, kcal/kg	3100	3100	3100	3100
Calcium, %	0.87	0.87	0.87	0.87
Na, %	0.18	0.18	0.18	0.18
K, %	0.98	0.99	1.00	1.01
Cl,%	0.26	0.26	0.26	0.26
Total Phos, %	0.69	0.71	0.73	0.75
Avail Phos %	0.44	0.44	0.45	0.45
Cysteine, %	0.35	0.35	0.35	0.35
Lysine, %	1.37	1.36	1.35	1.35
Methionine, %	0.57	0.57	0.57	0.57
TSAA, %	0.88	0.88	0.88	0.88
Threonine, %	0.91	0.90	0.90	0.89
Crude Fiber, %	1.73	1.78	1.83	1.87
Linoleic acid, %	1.36	1.32	1.27	1.23
**Analyzed compositions**				
Dry matter, %	91.01	91.33	90.83	91.38
Crude protein, %	21.85	21.63	21.80	21.74
Crude Fiber, %	1.32	1.54	1.22	1.72
Ash,%	6.33	5.95	5.85	6.2

Abbreviation: CP, crude protein; ME, Metabolisable energy; Phos, Phosapate; TSAA, total sulfur amino acid.

a Provided per kg of diet: Protein, 18%; CaCO_3_, 19%; Monocalcium phosphate, 7%; NaCl, 4.5%; Methionine, 2%; Lysine, 4%; Threonine, 1%; Choline chloride, 1%; Iron (sulphate), 2800 mg; Zinc (oxide), 2480 mg; Manganese (oxide), 3600 mg; Copper (sulphate), 240 mg; Iodine (IK), 60 mg; Selenium (SeNa), 14 mg; Cobalt (sulphate), 14 mg; Magnesium (oxide), 800 mg; Antioxidant (BHT), 32 mg; Vitamin A, 420,000 IU; Vitamin D_3_, 80,000 IU; Vitamin E, 800 mg; Vitamin C, 40 mg; Vitamin K_3_, 80 mg; Vitamin B_1_, 80 mg; Vitamin B_2_, 200 mg; Vitamin B_6_, 80 mg; Vitamin B_12_, 0.6 mcg; Biotine, 10.4 mg; Folic acid, 20 mg; Nicotinic acid, 400 mg; Pantothenic acid, 160 mg.

**Table 3:** Ingredients and nutrients composition of experimental diets (26–35 days).

Ingredient (%)	Control	Herbal Mixture (%)		
		2.5	5	7.5
Corn grain	59.56	57.70	55.78	53.86
Soy bean meal (41% CP)	34.94	34.32	33.70	33.09
Soybean oil	1.30	1.32	1.37	1.41
Dicalcium phosphate	0.32	0.30	0.27	0.24
CaCO_3_	0.97	0.95	0.96	0.97
Common Salt	0.22	0.22	0.22	0.21
Premix concentrate^a^	2.50	2.50	2.50	2.50
DL-Methionine	0.16	0.17	0.17	0.17
Herbal mixture	0.00	2.50	5.00	7.50
**Calculated analysis**				
Crude protein, %	19.5	19.5	19.5	19.5
ME, Kcal/kg	3200	3200	3200	3200
Calcium, %	0.79	0.78	0.78	0.78
Na, %	0.16	0.16	0.16	0.16
K, %	0.87	0.89	0.90	0.91
Cl, %	0.23	0.23	0.23	0.23
Total Phos, %	0.63	0.64	0.65	0.67
Avail Phos, %	0.39	0.39	0.39	0.39
Cysteine, %	0.32	0.32	0.32	0.32
Lysine, %	1.21	1.21	1.20	1.19
Methionine, %	0.52	0.52	0.52	0.52
TSAA, %	0.80	0.80	0.80	0.80
Threonine %	0.82	0.81	0.81	0.80
Crude Fiber, %	1.76	1.81	1.86	1.90
Linoleic acid, %	2.11	2.08	2.06	2.04
**Analyzed compositions**				
Dry matter, %	91.19	90.81	90.95	91.12
Crude protein, %	19.54	19.73	19.66	19.59
Crude Fiber, %	1.88	1.44	1.76	1.15
Ash,%	5.90	5.91	5.41	5.79

Abbreviation: CP, crude protein; ME, Metabolisable energy; Phos, Phosapate; TSAA, total sulfur amino acid.

a Provided per kg of diet: Protein, 18%; CaCO_3_, 19%; Monocalcium phosphate, 7%; NaCl, 4.5%; Methionine, 2%; Lysine, 4%; Threonine, 1%; Choline chloride, 1%; Iron (sulphate), 2800 mg; Zinc (oxide), 2480 mg; Manganese (oxide), 3600 mg; Copper (sulphate), 240 mg; Iodine (IK), 60 mg; Selenium (SeNa), 14 mg; Cobalt (sulphate), 14 mg; Magnesium (oxide), 800 mg; Antioxidant (BHT), 32 mg; Vitamin A, 420,000 IU; Vitamin D_3_, 80,000 IU; Vitamin E, 800 mg; Vitamin C, 40 mg; Vitamin K_3_, 80 mg; Vitamin B_1_, 80 mg; Vitamin B_2_, 200 mg; Vitamin B_6_, 80 mg; Vitamin B_12_, 0.6 mcg; Biotine, 10.4 mg; Folic acid, 20 mg; Nicotinic acid, 400 mg; Pantothenic acid, 160 mg.

### 2.4 Virus inoculation

All chickens were inoculated with 0.5 mL CIAV (Cux-1 strain) suspension containing 10^6.6^ median tissue culture infective doses using the oral route at seven days of age. The cux-1 virus was grown in MDCC-MSB1 cells [34].

### 2.5 Performance and carcass traits measurements

The experiment recorded the body weight (BW) and feed intake (FI) on days 10, 25, and 35. These measurements were used to calculate the weight gain (WG) and feed conversion ratio (FCR). In addition, the mortality and weight of the birds were recorded during the experiment to adjust for the FCR. One bird per cage was randomly selected and slaughtered at 25 days of age. The weights of carcass parts (breast, thigh, and drumstick muscle), internal organs (crop, proventriculus, gizzard, pancreas, liver, spleen, heart, bursa of Fabricius, and abdominal fat), and the weight and length of intestinal segments (duodenum, jejunum, ileum, and caeca) were recorded using a digital balance with an accuracy of 0.01 g at 25 days of age. All weight and length data are expressed as percentages of live BW [35].

### 2.6 Blood and serum collection

The blood samples were collected from a brachial vein of selected birds for slaughtering at 25 days of age, centrifuged at 3500 rpm for 15 min to separate serum, and stored at −20 °C until analysis. Commercial kits (Biolabo S.A.S, Les Hautes Rives, 02160 Maizy, France) were used to measure total serum cholesterol, triglycerides, very-low-density lipoprotein (VLDL), high-density lipoprotein (HDL), glucose, total protein, and uric acid. Blood samples were stored in tubes containing ethylenediaminetetraacetic acid (EDTA, 2 mg/mL blood) [36] to measure hematological parameters, including red blood cell count (RBC), hematocrit (HCT), hemoglobin (Hb), mean corpuscular volume (MCV), mean corpuscular hemoglobin (MCH), mean corpuscular hemoglobin concentration (MCHC). Additionally, the differential white blood cell (WBC) count, which included lymphocyte, MID, granulocyte, heterophile, and lymphocyte-to-heterophile ratios, was evaluated. A veterinary hematology analyzer (SmartVet) was used to determine the heterophile to lymphocyte ratio (H/L); the number of heterophils was divided by the number of lymphocytes according to Lentfer et al. [36].

### 2.7 Intestinal morphology

At 25 days of age, approximately 4 cm of the middle of the duodenum, jejunum, and ileum were removed, washed with physiological serum, and fixed in 10% formalin for morphometric analysis. The automatic tissue processor dehydrated, clarified, and immersed the tissue in paraffin. The melted paraffin wax was molded using a paraffin dispenser. Sections (5 mm) were cut from the waxed tissue using a microtome (Model Sakura Finetek Europe BV, Alphen aan den Rijn, Netherlands) and mounted on glass slides using pre-warmed water (50 °C). The slides were stained with hematoxylin and eosin. Morphometric traits, such as villus height (VH), crypt depth (CD), villus height to crypt depth ratio (VH/CD), and villus surface area (SA), were determined at a magnification of 40X using a light microscope. The mean value of 10 villi per sample was used for further analysis [37].

### 2.8 Hepatic histopathology

Approximately 1 cm^3^ of the left lobe of the liver was taken from the slaughtered birds and fixed by immersion in 10% buffered formalin until further analysis. The steps of tissue processing, molding in paraffin, cutting tissue sections, and staining were done as described in Section 2.7. Fat vacuole contents of liver slides, indicating liver steatosis, were graded according to their severity using the following scale: 0 (0–19%), 1 (20–39%), 2 (40–59%), 3 (60–79%), and 4 (80–100%) at 40X magnification [38]. Additionally, the level of leukocyte infiltration was measured and scored as the follows: 0 indicating no damage, 1 indicating mild damage, 2 indicating moderate damage, and 3 indicating severe damage [39].

### 2.9 Liver enzyme activity

Commercial kits from Biolabo S.A.S., Les Hautes Rives, 02160 Maizy, France, were used to measure serum aspartate aminotransferase (AST), alanine transaminase (ALT), and alkaline phosphatase (ALP) [40].

### 2.10 Statistical analysis

All obtained data were subjected to analysis of variance using General Linear Model procedures from the SAS Institute [41] (SAS 9.1, Carey, NC). The individual bird sample was the experimental unit for all parameters other than performance and mortality, whereas the cage served as the experimental unit for the performance trait analysis. Once the model was considered significant, the mean differences were assessed using Tukey's test (P < 0.05).

## 3 Results

### 3.1 Herbal mixture analysis

Table 4 shows the chemical composition of the HM, including crude protein, crude fiber, ether extract, nitrogen-free extract, and ash, all of which are presented on a dry matter (DM) basis.

**Table 4:** Chemical composition of herbal mixture on a DM basis (%).

Parameters	DM	Ash	CP	CF	EE	NFE
**Herbal Mixture**	91.70	4.10	16.00	3.82	3.49	64.29

Abbreviation: CF, Crude fibre; CP, Crude protein; DM, Dry matter; EE, Ether extract; NFE, Nitrogen-free extract.

### 3.2 Bioactive components of herbal mixture extract

**Fig. 1:**
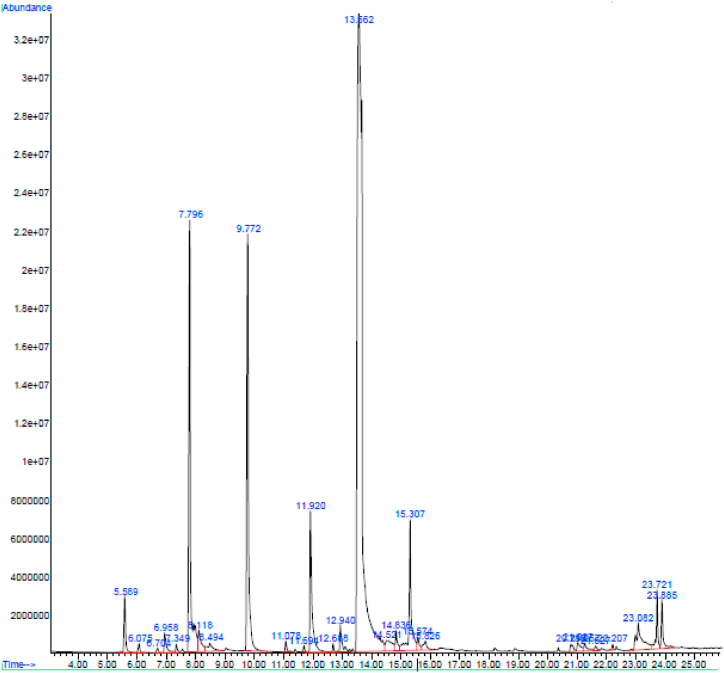
Chromatographic profile (GC-MS) of the herbal mixture ethanolic extract.

The bioactive components of the herbal mixture were identified using GC-MS analysis. The chromatogram of the ethanolic extract of the herbal mixture revealed many peaks. The five most-abundant components are listed in Fig. 1 and Table 5. Among all chemical components, the most expressed were anethole (52.98%), d-limonene (11.20%), fenchone (11.19%), α-longipinene (4.58%), and estragole (4.48%). These five compounds are all monoterpenes or phenylpropene, which according to the literature, are the most antimicrobial, antibacterial, antioxidant, and antiviral agents in herbal medicines.

**Table 5:** Pharmacological activities of the five most expressed bioactive compounds in herbal mixture ethanolic extract.

R. time (minute)	Compound name	Com. %	Pharmacological activity	References
13.563	Anethole	52.980%	Antiviral and antimicrobial activity	[89,90]
7.796	D-Limonene	11.202%	Antimicrobial, antibacterial, and antiviral activity	[91,92]
9.772	Fenchone	11.188%	Antiviral and antibacterial activity	[93,94]
15.307	α-Longipinene	4.585%	Antimicrobal, antifungal, and antioxidant activity	[95,96]
11.921	Estragole	4.480%	Antiviral and antimicrobal activity	[97,98]

Abbreviation: R. time, Retention time; Com., compound.

### 3.3 Growth performance

The effects of different levels of HM (0, 2.5, 5, and 7.5%) on BW and WG in inoculated broiler chicks from 1 to 35 days of age are presented in Table 6. Birds fed 2.5 and 5% of the HM significantly showed higher live BW compared to the group fed 7.5% of the HM (P < 0.05) but were numerically different from the control group. Experimental diets did not significantly affect broiler BW at 25 days of age. Diets containing 5 and 7.5% HM resulted in reduced BW compared to that of the control and 2.5% HM at 35 days of age (P > 0.05). In other words, higher doses of HM significantly (P < 0.05) reduced BW. The chicks that consumed 2.5 and 5% of HM showed maximum WG compared to 7.5% HM during 1–10 days means the inclusion of different levels of HM in basal diets had no significant effect on WG (P > 0.05) compared to the control. Diets containing different levels of HM had no significant effect on WG at 11–25 days of age. In contrast, the WG of the various groups varied significantly throughout the 26–35 days of age and the entire rearing period (1–35 days of age). Birds fed 7.5% HM showed the lowest WG compared to the control, and birds fed 2.5% HM at 26–35 days of age (P < 0.05). Birds fed 7.5% HM exhibited lower WG than other groups at 1–35 days of age (P < 0.05). In general, the birds that received 2.5% HM showed the highest WG at 26–35 and 1–35 days of age.

**Table 6:** Effects of herbal mixture (HM) on body weight (g), weight gain (g), feed intake (g), feed conversion ratio (g/g), and mortality (%) in broiler chicken inoculated by CIAV at 7 days of age.

Performance	Treatments	Control	2.5%HM	5%HM	7.5%HM	Probability	SEM
Body weight	10 day	251^ab^	263^a^	264^a^	243^b^	0.004	2.833
	25 day	1084	1019	1066	1086	0.530	16.8
	35 day	1833^a^	1852^a^	1750^b^	1615^c^	0.0003	41.6
Weight gain	1–10 day	213^ab^	225^a^	226^a^	205^b^	0.005	2.80
	11–25 day	840	767	785	819	0.300	14.8
	26–35 day	749^a^	810^a^	684^ab^	532^b^	0.014	33.8
	1–35 day	1809^a^	1815^a^	1720^b^	1560^c^	0.045	24.5
Feed intake	1–10 day	286	269	286	288	0.1433	3.41
	11–25 day	1174	1126	1229	1264	0.3656	27.6
	26–35 day	1010^b^	1306^a^	1052^b^	810^c^	0.0001	48.5
	1–35 day	2470^bc^	2701^a^	2567^ab^	2362^c^	0.0003	83.1
Feed conversion ratio	1–10 day	1.30^b^	1.22^b^	1.28^b^	1.42^a^	0.0001	0.02
	11–25 day	1.40	1.47	1.51	1.44	0.4874	0.026
	26–35 day	1.54	1.68	1.51	1.64	0.3204	0.038
	1–35 day	1.39^b^	1.48^ab^	1.51^a^	1.51^a^	0.0030	0.032
Mortality	11–25 day	27.50^a^	10.74^b^	23.33^ab^	10.00^b^	0.0103	2.70
	26–35 day	23.0^a^	20.2^ab^	11.5^bc^	4.40^c^	0.0010	2.14
	1–35 day	50.5^a^	30.94^ab^	24.85^ab^	14.4^b^	0.0007	3.43

^a–c^Means in the same row with different letters are different (P < 0.05).

Abbreviation: CIAV, Chicken infectious anemia virus.

The effects of experimental diets on the FI and FCR of inoculated broilers from 1 to 35 days of age are presented in Table 6. There were no differences in FI between the experimental groups regarding FI during 1–10 and 11–25 days of age (P > 0.05). Nevertheless, birds fed 2.5% HM had increased FI compared to that of the birds that received 7.5% HM at 26–35 and 1–35 days of age (P < 0.05). FCR for birds fed 2.5% of the HM was the lowest among all treatments numerically during 1–10 days of age, and were significantly different compared to birds that received 7.5% of the mixture, which had the highest FCR. There were no differences in FCR among all treatments at 11–25 or 26–35 days of age. The FCR increased gradually with increasing HM levels in the broiler chicken diet throughout the rearing period (P < 0.05).

### 3.4 Mortality

The effects of HM on the mortality rate of broiler chickens inoculated with CIAV are presented in Table 6. The mortality rate of chickens was zero at 1–10 days of age; however, there were differences after inoculating chicks with CIAV at 11–25 days of age. The mortality rate was reduced in the 2.5 and 7.5% HM supplementation compared to that of the control at 11–25 days of age (P < 0.05). The mortality rate of birds fed diets containing 5 and 7.5% of the HM was lower than that of the control at 26–35 days of age (P < 0.05). The mortality rate was considerably lower in birds that consumed different levels of HM than in the control group at 1–35 days of age (P < 0.05). The increase in HM levels gradually decreased the mortality rate during the total rearing period (P < 0.05), such that birds fed 7.5% of the HM had the lowest mortality rate.

### 3.5 Carcass traits and internal organs

The effects of HM on the relative weights of the breast, thigh, and drumstick muscles in live BW (g/100 g) are presented in Table 7. There were no significant differences among treatments (P > 0.05). The effects of the experimental diets on the relative weight of internal organs to live BW (g/100 g) at 25 days of age are presented in Table 7. There were no significant differences for the crop, gizzard, pancreas, liver, or heart (P > 0.05). However, the birds fed 7.5% HM had the lowest weight of the proventriculus compared to the other groups (P < 0.05). Diets containing 2.5 and 5% HM resulted in a significantly decreased spleen weight compared to the control (P < 0.05). The bursa of Fabricius in birds that consumed 7.5% of the HM were significantly heavier than those of the other groups. Additionally, the birds that received 5 and 7% HM had the lowest abdominal fat weights (P < 0.05).

**Table 7:** Effects of herbal mixture (HM) on relative weight and length of carcass segments and internal organs to live body weight (g/100g) in broiler chicken inoculated by CIAV at 7 days of age.

Treatments	Control	2.5%HM	5%HM	7.5%HM	Probability	SEM
**Weight**						
Carcass	64.88	64.87	65.15	64.33	0.8318	0.303
Breast	21.04	20.47	20.40	19.93	0.6424	0.287
Thigh	4.77	4.57	4.73	4.77	0.8878	0.099
Drumstick	4.58	4.27	4.20	4.22	0.0823	0.062
Crop	0.66	0.51	0.48	0.47	0.081	0.032
Proventriculus	0.69^a^	0.67^a^	0.55^ab^	0.52^b^	0.047	0.027
Gizzard	1.28	1.35	1.29	1.36	0.844	0.033
Pancreas	0.29	0.29	0.35	0.33	0.153	0.012
Liver	2.41	2.63	2.55	2.54	0.271	0.037
Spleen	0.14^a^	0.09^b^	0.09^b^	0.11^ab^	0.021	0.006
Heart	0.47	0.48	0.50	0.47	0.533	0.008
Bursa of fabriciuos	0.20^ab^	0.17^b^	0.19^b^	0.24^a^	0.005	0.008
Abdominal fat	0.62^ab^	0.86^a^	0.44^b^	0.54^b^	0.008	0.052
Duodenum	0.84^b^	1.01^ab^	1.06^a^	0.97^ab^	0.0258	0.025
Jejunum	3.04^b^	3.60^a^	3.76^a^	3.64^a^	0.0001	0.071
Ileum	2.66	2.15	2.53	2.59	0.1814	0.085
Cecum	0.93	1.00	0.94	0.79	0.4751	0.047
**Length**						
Duodenum	2.05	1.97	2.25	1.99	0.2132	0.053
Jejunum	5.09	4.89	5.27	5.62	0.3399	0.150
Ileum	4.69	4.38	5.09	4.61	0.2705	0.131
Cecum	1.31	1.22	1.40	1.20	0.2546	0.038

^a-b^Means in the same row with different letters are different (P < 0.05).

Abbreviation: CIAV, Chicken infectious anemia virus.

The effects of the experimental diets on the relative weights and lengths of the duodenum, jejunum, ileum, and cecum are shown in Table 7. There were no statistically significant differences among the experimental groups regarding the relative weights of the ileum and cecum or the relative lengths of the duodenum, jejunum, ileum, and cecum (P > 0.05). The relative weight of duodenum was the highest in the birds that received 5% of the HM (P < 0.05). Different levels of HM supplementation increased the relative jejunal weight of chickens compared to those of the control birds (P < 0.05).

### 3.6 Serum biochemical traits

Table 8 shows the significant differences in serum cholesterol, triglyceride, and VLDL levels. Supplementation with 5% of the HM decreased triglyceride and VLDL concentrations compared with that of the control, which showed the highest concentration. An increase in HM levels gradually decreased total cholesterol levels, especially at 7.5%, significantly affecting total cholesterol levels compared with that of the control group (P < 0.05). The results also showed no significant differences in serum HDL, glucose, creatinine, uric acid, or total protein concentrations among the treatments.

**Table 8:** Effects of herbal mixture (HM) on serum biochemical, liver function enzymes and histomotphology in broiler chicken were inoculated by CIAV at 7 days of age.

Treatments	Control	2.5%HM	5%HM	7.5%HM	Probability	SEM
Cholesterol (mg/dL)	68.88^a^	66.71^ab^	65.33^ab^	61.92^b^	0.0235	5.36
Triglyceride (mg/dL)	50.91^a^	36.81^ab^	22.88^b^	47.42^a^	0.0037	3.48
VLDL (mg/dL)	10.18^a^	7.36^ab^	6.03^b^	9.48^ab^	0.0317	0.595
HDL (mg/dL)	49.5	50.7	53.9	54.6	0.8627	2.26
Glucose (mg/dL)	217	206	214	226	0.5964	4.96
Creatinine (mg/dL)	0.278	0.217	0.250	0.358	0.1564	0.022
Uric acid (mg/dL)	5.93	5.04	5.24	4.33	0.1302	0.235
Total protein (mg/dL)	2.81	2.86	2.61	2.89	0.6514	0.082
ALT (U/L)	26.44	25.84	26.05	26.58	0.7176	0.24
AST (U/L)	194	200	208	209	0.5074	3.99
ALP (U/L)	928^a^	484^b^	458^b^	442^b^	0.0001	8.5
Steatosis	3.00^a^	3.17^a^	2.83^a^	0.75^b^	0.0158	0.293
Leukocyte infilteration	Mild	Mild	Mild	Low	0.1750	0.200

^a-b^Means in the same row with different letters are different (P < 0.05).

Abbreviation: ALP, Alkaline phosphatase; ALT, Alanine transaminase; AST, Aspartate transaminase; CIAV, Chicken infectious anemia virus.

### 3.7 Enzymes activity and histopathology of liver

As shown in Table 8, liver enzyme and histomorphology results revealed no significant differences in liver ALT and AST levels among the treatments. Diets supplemented with different levels of HM significantly decreased liver ALP levels compared to that of the control (P < 0.05). Histopathological analysis of the liver revealed that supplementation with 7.5% HM significantly decreased liver fat vacuoles compared with that of other groups (P < 0.05). Histopathological analysis of the liver revealed that birds fed diets containing 7.5% HM had lower infiltration of leukocytes around the hepatocytes compared to that of other groups with mild infiltration.

### 3.8 Hematological parameters

All estimated hematological parameters, such as RBC, HCT, HB, MCV, MCH, and MCHC of the CIAV-infected groups, are summarized in Table 9. No significant differences in RBC and MCH (P > 0.05) were observed between groups. Various levels of HM in diets resulted in significant increases in HCT and Hb values (P < 0.05). Birds fed 2.5% of the HM showed maximum values for HCT and Hb. Including 2.5% and 7.5% HM in diets resulted in a significant increase in MCV compared to that of the control group (P < 0.05). In contrast to other CIAV-infected groups, the chicks fed 7.5% of HM exhibited the greatest MCV value. The control group had a higher MCHC than the HM group (P < 0.05).

**Table 9:** Effects of herbal mixture (HM) on hematological parameters and different leukocyte types in broiler chicken inoculated by CIAV at 7 days of age.

Treatments	Control	2.5%HM	5%HM	7.5%HM	Probability	SEM
RBC ( × 10^6^ cell/μL)	2.99	4.03	3.94	3.78	0.074	0.158
HCT (%)	25.20^b^	38.25^a^	33.50^a^	33.50^a^	0.001	1.48
Hb (g/dL)	10.56^b^	14.47^a^	13.91^a^	13.05^a^	0.0009	0.463
MCV (fL)	70.0^b^	87.1^a^	82.2^ab^	88.6^a^	0.014	2.30
MCH (pg/cell)	30.07	34.40	35.84	32.41	0.061	0.841
MCHC (%)	42.89^a^	37.63^b^	38.70^b^	38.61^b^	0.0001	0.510
Lymphocyte (%)	64.58^ab^	66.65^a^	61.25^b^	61.25^b^	0.0244	0.78
Mid (%)	6.00^b^	12.05^a^	11.20^a^	7.46^ab^	0.0055	0.79
Granulocyte (%)	34.45^b^	34.70^b^	37.94^a^	38.75^a^	0.0288	0.68
Heterophile (%)	31.25^a^	17.12^b^	30.46^a^	32.15^a^	0.0001	1.68
L/H	2.05^b^	3.69^a^	2.27^b^	1.93^b^	0.0001	0.19


^a-b^Means in the same row with different letters are different (P < 0.05).

Abbreviation: CIAV, Chicken infectious anemia virus; RBC, Red blood; HCT, Hematocrite; Hb, Hemoglobine; MCV, Mean corpuscular volume; MCH, Mean corpuscular hemoglobin; MCHC, Mean corpuscular hemoglobin concentration; Mid, Sum of basophiles, monocytes, and eosinophiles; L/H, Lymphocytes to heterophiles ratio.

### 3.9 White blood cells

Table 9 shows the effects of HM supplementation on white blood cell counts, including lymphocytes, MID, granulocytes, heterophiles, and lymphocyte to the heterophile ratio (L/H). Results indicated that the lymphocyte count was lower (P < 0.05) in birds fed with 5% and 7.5% HM compared to that of the birds fed with 2.5% of HM, but there was no significant difference compared to that of the control group. MID was higher in birds consuming 2.5 and 5% HM compared the that of the control (P < 0.05). The highest percentage of MID was observed in birds fed by 2.5% HM. Granulocytes were higher (P < 0.05) in the groups that consumed 5 and 7.5% of the HM compared to the groups fed control and 2.5% of the HM. Birds receiving 2.5% of the HM in their diet had lower (P < 0.05) heterophile than other groups. Supplementation of the bird’s diet with 2.5% of the HM significantly increased the L/H ratio compared to other groups (P < 0.05).

### 3.10 Intestinal morphology

The effects of HM supplementation on the intestinal histomorphology of broilers are shown in Table 10. There were no significant differences in the duodenum, jejunum, and ileum morphology indices (VH, CD, VH/CD, and SA) among the treatments (P > 0.05).

**Table 10:** Effects of herbal mixture (HM) on villus height (VH), crypt depth (CD), villus height to crypth depth ratio (VH/CD) and villus surface area (SA) in broiler chicken were inoculated by CIAV at 7 days of age.

Intestine segment	Treatments	Control	2.5%HM	5%HM	7.5%HM	Probability	SEM
**Duodenum**	VH (μm)	964	1140	1060	726	0.476	83.22
	CD (μm)	177	194	226	244	0.502	15.90
	VH/CD	5.90	5.92	4.89	3.02	0.402	0.61
	SA (mm^2^)	0.12	0.14	0.15	0.09	0.694	0.014
**Jejunum**	VH (μm)	800	632	576	750	0.194	43.63
	CD (μm)	211	206	183	214	0.767	10.62
	VH/CD	3.79	3.17	3.15	3.51	0.422	0.15
	SA (mm^2^)	0.09	0.07	0.07	0.08	0.477	0.007
**Ileum**	VH (μm)	651	754	562	530	0.693	61.13
	CD (μm)	158	172	174	212	0.215	9.77
	VH/CD	4.07	4.28	3.31	2.48	0.343	0.37
	SA (mm^2^)	0.06	0.08	0.07	0.06	0.748	0.006

^a–c^Means in the same column with different letters are different (P < 0.05).

Abbreviation: CIAV, Chicken infectious anemia virus.

## 4 Discussion

Before the chicks were challenged with CIAV during the starter phase (1–10 days), the BW and WG were slightly higher in the 2.5% and 5% HM groups than the control group and significantly higher in the 7.5% HM group. The low-dose inclusion of HM resulted in a reduction in FCR, indicating that a lower level of HM enhanced growth.

Birds were challenged with CIAV during the grower phase (11–25 days), and chicken performance was unaffected by adding various levels of HM to their diet. However, in the finisher phase (26–35 days), HM supplementation considerably decreased FI, except in the 2.5% group. This decrease in FI reduced BW and WG compared to the control and 2.5% treatments but did not affect the FCR. Supplementation with high levels of HM in the diet of broilers negatively influenced FI, BW, WG, and FCR. This is in line with previous studies showing that adding a low dose of garlic, onions, and apple cider vinegar to broiler diets did not affect performance [14,42,43]. According to previous studies, the phytogenic compounds in garlic, onion, and apple cider vinegar are characterized by high concentrations of organosulfur compounds and organic acids. These compounds improve the performance of birds by stimulating the release of digestive enzymes that improve digestion and nutrient absorption, thereby improving the growth performance of broilers [44,45]. However, at high doses, it negatively affects performance [46,47].

The reduction in FI may be due to the high concentration of aromatic compounds in onions and garlic, which decrease feed palatability [47]. However, the reasons for the adverse effects of garlic and onions on performance, mainly on FI, require further clarification and research.

HM contains garlic and onion, two*Allium* genus members with antiviral, antibacterial, and hypolipidemic properties. The most critical components of the *Allium* genus are sulfuric compounds with antibacterial and antiviral effects. These effects increase resistance to CIAV and other infections because of the reduced adverse effects of CIAV on hematological parameters, leukocytes, liver function enzymes, and other prior factors. Consequently, mortality decreased drastically with increasing HM doses.

The relative weights of internal organs, including the crop, gizzard, pancreas, liver, and heart, to BW were unaffected by HM supplementation. The results of studies by Kharde et al. [48], Lee et al. [49], Allahdo et al. [50], and Omar et al. [51] concur with our findings. The relative weight of the proventriculus decreased when HM was administered at higher doses; this relative weight reduction may be related to a decline in feed consumption. Atrophy or a reduction in organ size may be due to a reduction in metabolism or organ activity. The reduction in the relative weight of the spleen as a result of HM supplementation may be due to the antimicrobial and antiviral effects of garlic and onions. This finding agrees with those of Borgohain et al. [52], Ismail et al. [53], and Martínez et al. [54], who found that the spleen weight of inoculated broilers decreased when acetic acid and garlic were added to their diets. This finding indicated that supplementing broiler diets with HM did not affect the relative weight of the bursa of Fabricius or abdominal fat compared to that of the control.

The relative weight of duodenum was considerably higher due to adding 5% HM to inoculated broilers' diet. In addition, dietary supplementation with different levels of HM increased the relative weight of the jejunum. As investigated by researchers, onion and garlic in the stomach increase acid secretion through the activation of the chief cell or parietal cells via a cholinergic mechanism [55]. Adding garlic and onion to the diet increases the production of bile acids and pancreatic juice [56]. We included apple cider vinegar, which has acidic properties, in the herbal preparations. The combination of these factors increases intestinal acidity and enlarges the mucosal layer of the digestive system. These events prevent the development of harmful bacteria [57], decrease the number of harmful bacteria, and damage the intestinal epithelial layer damage [58]. Reduced damage to the gut may result in intestinal WG.

HM supplementation reduced total cholesterol, triglyceride, and VLDL levels in chickens infected with CIAV compared to that of the control group. Sulfuric compounds are primarily found in onions and garlic, which oxidize thiol compounds, either free or combined with protein and NADPH [59]. HM decreased cholesterol, triglyceride, and VLDL concentrations in broiler chickens and the number of fat vacuoles in the liver by oxidizing the thiol molecules required for lipid synthesis. This cholesterol, triglyceride, and VLDL level reduction agrees with some preview results [60,61]. Some researchers have found that onions do not affect total cholesterol or HDL levels [43,62]. These treatments did not affect the serum biochemical traits of the hens infected with CIAV, such as glucose, creatinine, uric acid, or total protein levels. This finding is consistent with previous results [[63], [64], [65], [66]].

The discovery of CIAV-DNA genes in liver cells indicated that the liver is an organ that maintains live CIAV, similar to the bone marrow, spleen, and thymus [67]. According to Kabariya et al. [68], CIAV infection increases the blood levels of liver enzymes, such as AST, ALT, and ALP, in infected hens compared to uninfected hens. However, HM supplementation did not affect ALT and AST levels but reduced ALP (P < 0.05). According to Omer et al. [69], supplementing chicken diets with onion and garlic did not affect AST and ALT levels but reduced ALP levels.

Our results showed that inoculation of control broilers with CAIV reduced the RBC count and significantly decreased HCT, Hb, and PCV values. However, adding HM to the diet of inoculated broilers improved these hematological parameters. Wani et al. [70] reported that CAIV suppresses the immune system and reduces HCT and RBC counts. These effects could be related to the antiviral effects of the organosulfur compounds in garlic and onion. Garlic contains various organosulfur compounds, including l-cysteine sulfoxides, glutamyl-*l*-cysteine peptides, and more than 30 sulfuric compounds [71]. Alliin (S-allyl-L-cysteine sulfoxide) is a common organosulfur component of garlic [72]. Allin is promptly transformed into allicin by alliinases when raw garlic is chopped, minced, crushed, or chewed [72,73]. According to prior research, allicin exhibits antiviral activities [22,74], immunomodulatory properties [75], anti-inflammatory qualities [76], antioxidant capabilities [77], and other pharmacological properties [73].

Onions also contain organosulfur components, such as allicin, ribavirin, and quercetin, which have antiviral properties [78]. As a result, HM improves hematological parameters, reduces the detrimental effects of CAIV in hens, and aids in developing immunity against other infectious agents. In response, diets containing garlic and onion minimized the effects of CAIV on hematological parameters and assisted stem cells in producing an average number of RBCs.

CIAV directly affects primary lymphoid organs, particularly precursor cells, and decreases lymphocyte levels [79]. The immune system is suppressed by reducing the production of WBCs in the bone marrow and developing T-cells in the thymus [80]. CIAV also suppresses bactericidal activity, expression of Fc receptors, releasing IL-1 in macrophages, and phagocytosis [81,82]. T and B lymphocyte formation, activation, and proliferation are reduced when IL-1 levels decrease [83]. Garlic and onion reduce the effects of CIAV in inoculated chickens through their antiviral properties present in them, such as inhibition of binding, fusion, entry of viruses into host cells, and inhibition of virus duplication and assembly [[20], [21], [22], [23], [24], [25], [26]]. At the same time, the attenuation of CIAV produces more WBCs in the bone marrow, especially lymphocytes, leading to the development of T-cells in the thymus. Garlic and onion increase the number of cells in the bone marrow and the proliferation of lymphocytes in the spleen and thymus [84,85]. HM supplementation significantly increased MID-cell and granulocyte numbers in chickens infected with CIAV. This result agrees with previous studies in which onion, garlic, and acetic acid improved lymphocyte and granulocyte counts and increased immunity in chickens [53,[86], [87], [88]]. Results showed that adding 2.5% HM to inoculated broilers' diet reduced heterophiles and increased the L/H ratio. Immune system activation upon viral exposure Administration of HM to inoculated chickens synergistically affected the immune system, leading to greater development and proliferation of lymphocytes than other immune cells in the innate immune system.

Our findings and those of previous studies showed that phytogenic ingredients (onion, garlic, and apple cider vinegar) contain various bioactive substances, including organosulfur compounds, flavonoids, fructans, fructooligosaccharides, and saponins. Consequently, their use as feed additives in poultry production is justified. Recent research has shown that using onion, garlic, and apple cider vinegar as a mixture in poultry diets significantly modulates growth performance, lipid metabolism, gut environment, and immunological responses, particularly under stress and disease conditions. This study will stimulate research on herbal mixtures as feed additives to control poultry health and disease.

## 5 Conclusion

Although adding high levels of HM (especially at the 7.5% level) to CIAV-infected broilers' diets reduced growth performance, it was clear that this negative effect was not present at the 2.5% level and even led to a slight increase in BW and WG in many cases. Interestingly, supplementation with different concentrations of HM reduced mortality throughout the rearing period. In addition to the hypolipidemic effects, the adverse effects of CIAV on HCT, Hb, MCV, and ALP levels were modulated by different concentrations of HM in infected chicks. However, adding 7.5% HM decreased liver fat vacuoles and infiltration of leukocytes around the hepatocytes significantly. Adding 2.5% of HM can potentially prevent the adverse effects of CIAV on hematological traits in broiler chicken flocks without adverse effects on performance.

## Author contribuition statement

Zana Azeez Abdulkareem conceived, designed, performed the experiments, and wrote the paper.Nihayat Ibrahim Mohammed contributed reagents, materials, analysis tools, or data. Asrin Abdollahi analyzed and interpreted the data and also wrote the paper. Omer Rasool Ahmed performed the experiments and contributed reagents, materials, analysis tools, or data. Osama Rahman Ghaffar analyzed and interpreted the data and wrote the paper. Hawkar Azad Khdir contributed reagents, materials, analysis tools, or data. Dashty Akram Salam performed the experiments, while Sarhang Ahmad Aziz, Mustafa Mama Mustafa, and Warzer Mohammed Mustafa also performed experiments. Zaniar Ali Abas and Omed Idrees Abid contributed reagents, materials, analysis tools, or data.

## Funding details

No organization provided funding for this paper.

## Data availability statement

You can access all the data by requesting it through the following link: https://docs.google.com/spreadsheets/d/1OW9T7-kCF-7720K9LeM6OjeJ6FCE8alP/edit?usp=sharing&ouid=111891967596185328820&rtpof=true&sd=true.

## Declaration of competing interest

The authors declare that they have no known competing financial interests or personal relationships that could have appeared to influence the work reported in this paper.
